# Rapid and Sensitive MicroRNA Detection with Laminar Flow-Assisted Dendritic Amplification on Power-Free Microfluidic Chip

**DOI:** 10.1371/journal.pone.0048329

**Published:** 2012-11-07

**Authors:** Hideyuki Arata, Hiroshi Komatsu, Kazuo Hosokawa, Mizuo Maeda

**Affiliations:** Bioengineering Laboratory, RIKEN, Wako, Japan; University of California, Merced, United States of America

## Abstract

Detection of microRNAs, small noncoding single-stranded RNAs, is one of the key topics in the new generation of cancer research because cancer in the human body can be detected or even classified by microRNA detection. This report shows rapid and sensitive microRNA detection using a power-free microfluidic device, which is driven by degassed poly(dimethylsiloxane), thus eliminating the need for an external power supply. MicroRNA is detected by sandwich hybridization, and the signal is amplified by laminar flow-assisted dendritic amplification. This method allows us to detect microRNA of specific sequences at a limit of detection of 0.5 pM from a 0.5 µL sample solution with a detection time of 20 min. Together with the advantages of self-reliance of this device, this method might contribute substantially to future point-of-care early-stage cancer diagnosis.

## Introduction

MicroRNAs (miRNAs) are short, highly conserved non-protein-coding single-stranded RNAs of typically 18 to 24 bases. MiRNAs repress gene expression in a sequence-dependent manner [1], and are associated with various human diseases including diabetes, Alzheimer’s, and cancer [2]–[5]. Thus, miRNAs have become increasingly important in determining disease diagnosis and prognosis. Some miRNAs circulate in human body fluid with an extremely low concentration at the early stage of cancer, and its expression profiling can detect or even classify cancer in the human body [6], [7]. Therefore, highly sensitive miRNA detection will open a new field of early-stage cancer diagnosis [2]–[9]. Various techniques such as quantitative polymerase chain reaction (qPCR), deep sequencing, and oligo microarrays are the major technical methods for miRNA profiling with their own strengths and drawbacks [10]. These techniques allow detection and quantification of miRNAs despite obstacles imposed by the intrinsic properties of miRNA, such as small size, wide range of melting temperature, and large number of highly homologous sequence variants [11]. Sequencing is considered the best choice for discovery of novel miRNAs and analysis of little variations, and qPCR the most reliable tool for sensitive quantification. The microarray method allows economic high-throughput profiling, but with low reliability [12]–[16]. It is prone to artificial errors and biases [17]. Considering point-of-care (POC) diagnosis, there are several additional requirements besides high sensitivity, such as a short detection time and small sample volume. These detection processes should be completed on a portable and compact device, which does not require huge equipment or trained personal for operation. Due to these specific requirements, none of the current techniques meets all the requirements for POC diagnosis.

**Figure 1:**
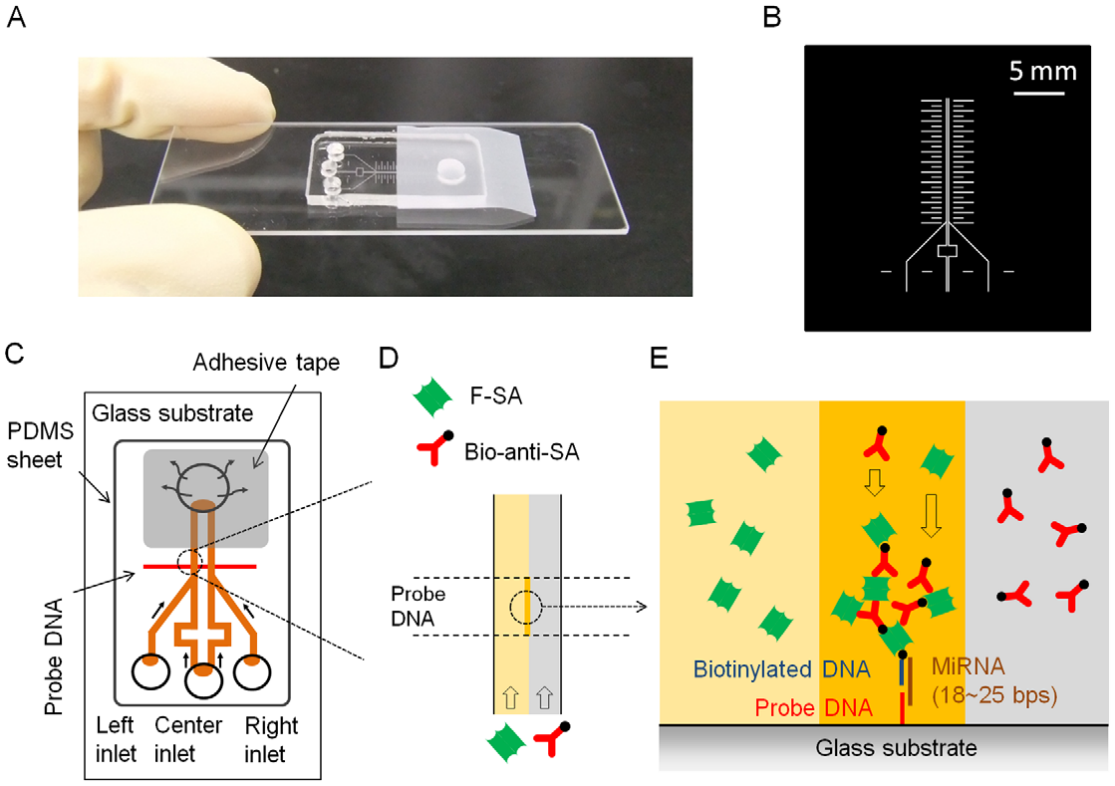
The microfluidic device, schematics of the sandwich hybridization, and laminar flow-assisted dendritic amplification (LFDA). (A) The microfluidic device. (B) The photomask used to fabricate the silicon mold. The ladder shaped pattern on both sides of the double-Y channels was designed as a ruler for general purposes. In this study, a part of it was used for alignment of the PDMS channel with the probe DNA pattern. The detours of the center channels were constructed to make the fluidic resistances, proportional to the channel length, uniform in all of the four branches. (C) Schematics of the power-free microfluidic device. PDMS absorbs air in the outlet chamber, thus being a self-stand pumping device. The width of the DNA probe pattern was 100 µm, and it was located 500 µm downstream of the confluent point of the Y-shaped channel. (D) Enlarged view of a laminar flow in the microchannel. F-SA and B-anti-SA are conveyed by the laminar flow. (E) Enlarged cross-sectional view of the sandwich hybridization and LFDA. The dendritic amplification takes place at the intersection between the probe DNA patterned surface and the interface of the laminar flow. F-SA: FITC-labeled streptavidin, B-anti-SA: biotinylated anti-streptavidin.

Recently, so-called top-down technologies such as MEMS (Micro-Electro-Mechanical Systems) have enabled us to fabricate structures of a sub-micrometer scale and to integrate multiple functional units on a single chip with the capability of batch fabrication [18], [19]. MicroTAS (Micro-Total Analysis Systems), which are usually developed by MEMS technology, enables rapid, sensitive, and parallel biological or chemical analysis with small amounts of samples because of the merits of miniaturization [20]–[23]. In handling small amounts of samples on a microscale, this system realizes a short diffusion distance [24], [25], high interface-to-volume ratio [26], [27], and small thermal mass [28], [29]. The material cost and amount of waste can also be reduced. The microfluidic device is one of the attractive candidates of POC technology for miRNA detection since it can reduce the assay time by conveying sample RNA molecules to immobilized probe DNA continuously by a microchannel. We have recently achieved miRNA detection in 20 min from a small sample volume by introducing a power-free microfluidic device, which eliminates the need for external power sources for fluid pumping [30]. The power-free microfluidic device was originally invented by our group [31], and is driven by energy that is stored in degassed poly(dimethylsiloxane) (PDMS) in advance. However, the limit of detection (LOD) of the miRNA was 0.62 nM [30], which was far above the criteria for practical application, and further improvement of the LOD was an inevitable challenge. In this report, the LOD of miRNA detection by power-free microfluidic device was improved by 3 orders of magnitude to 0.5 pM, by adopting laminar flow-assisted dendritic amplification (LFDA) [32]. LFDA is a signal amplification method for microfluidic analysis of surface-bound molecules. In LFDA, two amplification reagents are supplied from laminar streams to the surface-bound molecules, and these reagents construct dendritic structures growing over time.

**Table 1:** Sequences of oligonucleotides used in this study.

Nucleic Acids	Sequence (from 5′ to 3′)
aminated probe DNA	NH_2_- TTT TTT TTT TTT TTT TCA ACA TCA GT
biotynylated probe DNA	CTG ATA AGC TA-biotin
target miRNA (miR-21)	UAG CUU AUC AGA CUG AUG UUG A
random miRNA	UGG UGC GGA GAG GGC CCA CAG U

DNAs were from Operon Inc. and RNAs were from Greiner Inc.

## Results and Discussion

### Power-Free Microfluidic Device

The PDMS microfluidic device was designed and fabricated as reported elsewhere [33], with an additional process of selective immobilization of the aminated probe DNA on a glass plate. This microfluidic device allows self-pumping by PDMS air absorption without connecting to an external power supply. Figure 1 shows the device (A), a photomask used to fabricate the silicon mold (B), schematics of the device (C), and a description of LFDA (D, E). The power-free pumping technique is based on gas solubility in PDMS [34]. At atmospheric pressure, PDMS contains a large amount of air, which can be evacuated in a vacuum. When the PDMS is brought back under atmospheric pressure, air dissolves into the PDMS again. This air re-dissolution reduces the pressure in the waste reservoir when the reservoir is sealed with a piece of adhesive tape and the inlets are plugged with the solution droplets. Therefore, the solutions are pulled by the reduced pressure in the reservoir. Incidentally, the degassing of the microfluidic device does not need to be performed at the time of the analysis because the degassed device can be preserved in a gas-tight condition [35].

**Figure 2:**
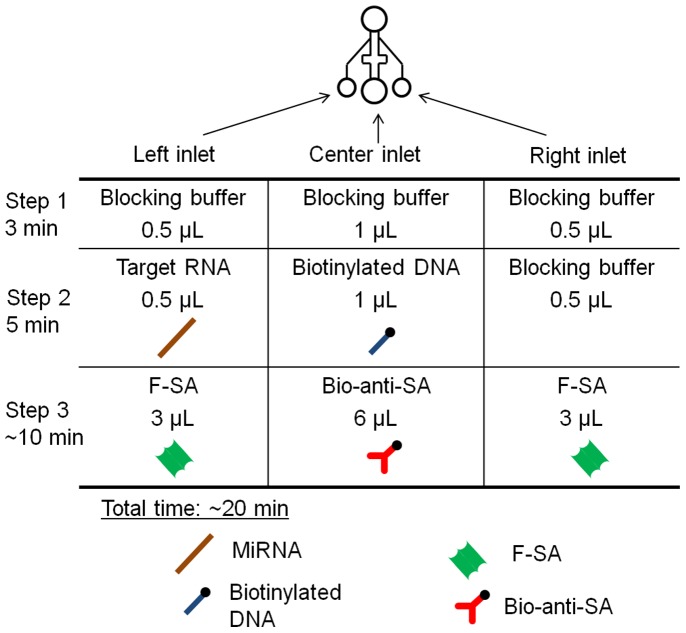
Schematic representation of the experimental protocol. The center channels were always used for one reagent at a time. Step 1: 0.5 µL of blocking buffer (BB) was injected from all the inlets and incubated for 3 min. Step 2: 0.5 µL of the target miRNA sustained in BB, 1 µL of biotinylated probe DNA (10 nM in the BB), and 0.5 µL of BB, were injected from the left, center, and right inlets, respectively. Step 3: After 5 min of incubation, 3 µL of F-SA (2.5 µg/mL in BB), 6 µL of B-anti-SA (25 µg/mL in BB), and 3 µL of F-SA (2.5 µg/mL in BB) were injected from the left, center, and right inlets, respectively.

**Figure 3:**
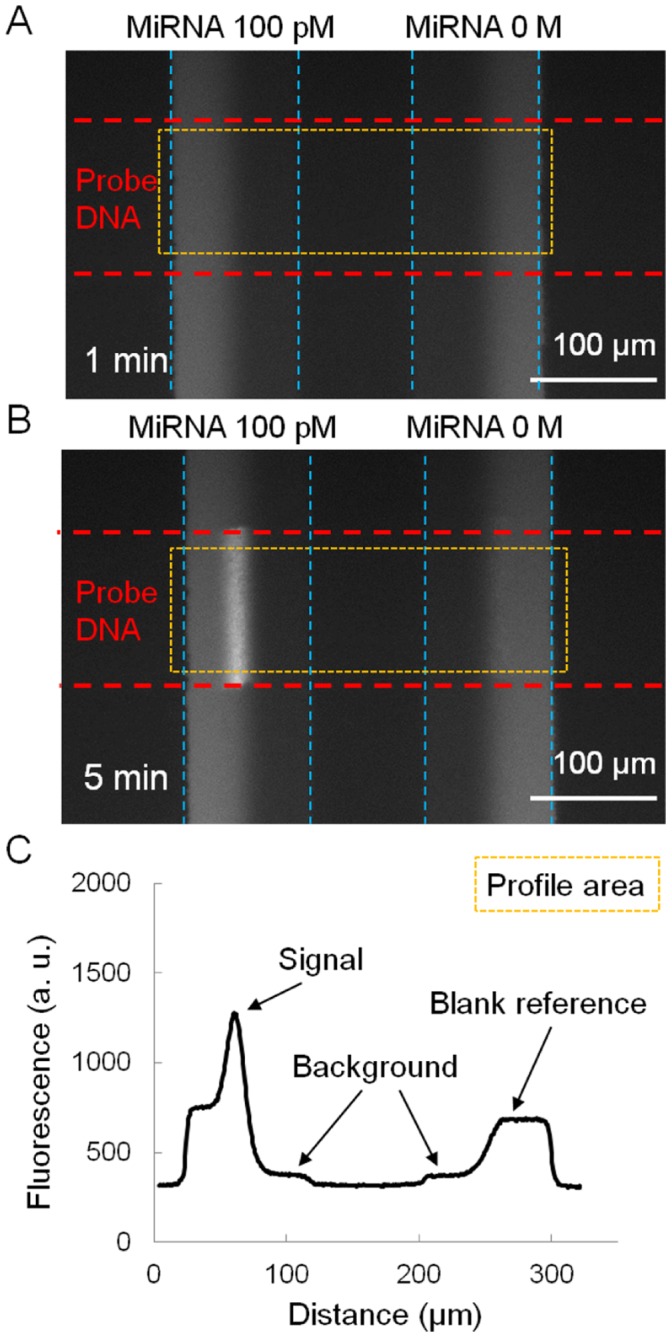
Fluorescent signals and their profiles. (A) Microscopic view of a fluorescent signal at an elapsed time of 1 min of LFDA. (B) Microscopic view of an amplified fluorescent signal at an elapsed time of 5 min of LFDA. The dendritic amplification is a three-phase reaction that takes place only at the intersection between the probe DNA patterned surface and the interface of the laminar flow. (C) Profile of the fluorescent intensity at an elapsed time of 5 min. In LFDA, the fluorescent signal appears in line. We defined the peak intensity in the left channel from the background level as the signal. Similarly, the maximum intensity in the right channel, which is indistinguishable from the fluorescence of bulk F-SA flow in this figure, was used as the blank reference.

Microfluidic channels prepared in this study were 100 µm in width and 25 µm in height. The microfluidic device had a pair of microchannels arranged in a ‘Y’ configuration. We adopted this double-Y design to carry out experiments in two different conditions simultaneously, one for the target miRNA detection and the other for a control blank reference. The double-Y channels were not completely independent; the center channels were always used for the same solution at a time. The aminated probe DNAs were patterned onto an aminated glass substrate via glutaraldehyde.

**Figure 4:**
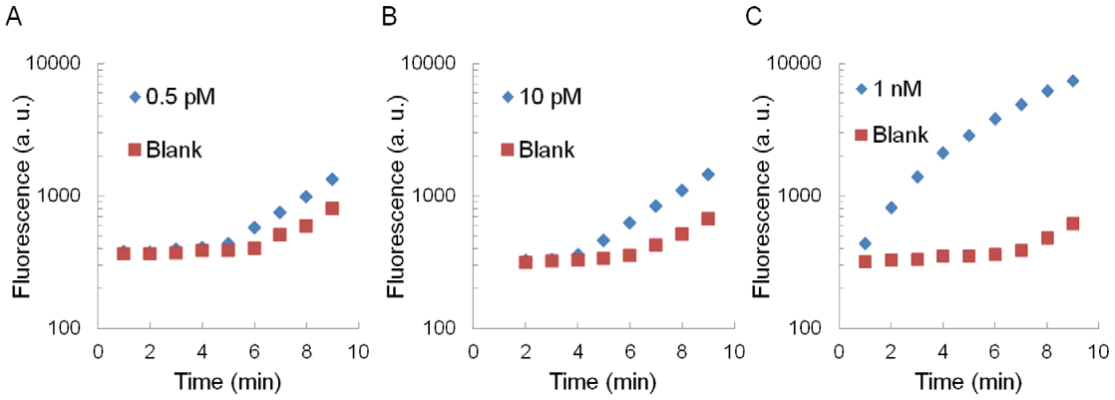
Time courses of amplified fluorescent signals in various miRNA concentrations and their blank references. 0.5 pM miRNA (A), 10 pM miRNA (B), and 1 nM miRNA (C). Note that the vertical axes are in logarithmic scale. Therefore the apparent linear increase in this figure means exponential increase in reality.

**Figure 5:**
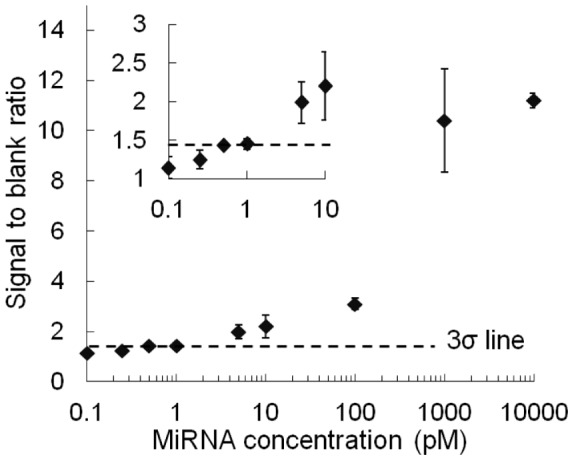
Signal to blank ratio vs. miRNA concentration. The inset shows an expanded view at low concentrations. The error bars represent standard deviations (*n* = 3). The “3σ line” was used for calculation of the LOD. This line was drawn at the position 3σ higher than the *y*-intercept of the fitting curve, where σ denotes the standard deviation of zero-concentration data (*n* = 5).

### MiRNA Detection and Signal Amplification

The sequences of oligonucleotides (probe DNAs and miRNAs) used in this study are listed in Table 1. MiR-21 was adopted as a model sequence since it is one of the most well-known miRNAs as a cancer marker [36], [37]. The signal was amplified after trapping the sample miRNA by sandwich hybridization. The sandwich construction was designed to take an advantage of the coaxial stacking effect [30], namely, when two contiguous tandem sequences are annealed on a same longer strand, base stacking at the nick enhances the hybridization stability and efficiency [38], [39]. For the signal amplification by LFDA, we used fluorescein isothiocyanate (FITC)-labeled streptavidin (F-SA) and biotinylated anti-streptavidin (B-anti-SA) as the amplification reagents. The two reagents were supplied from different microchannel branches to a common microchannel, where biotinylated probe DNA had been bound to the channel surface via the sandwich construct (Figure 1D, 1E). The use of laminar flow enabled rapid and continuous growth of aggregates of F-SA and B-anti-SA molecules on the sandwich construct. In our previous studies, we have found that mixing of the two reagents prior to application to the surface-bound target molecules has negative effect on the growth of the aggregates [32], [40].

Figure 2 shows use of the double-Y microchannels for miRNA detection and the LFDA procedure. Figures 3A and 3B show typical images of fluorescence microscopy with the target miRNA of 100 pM at an elapsed time of 1 min and 5 min, respectively, after the initiation of LFDA. A bright line appeared at the expected position; it appeared only on the area with the aminated probe DNA in the left channel in which the target miRNA had been supplied. This image proved that the LFDA successfully took place in a target-dependent manner. In addition, the bright line did not appear when the random miRNA of 10 nM ∼ 1 µM was injected, and thus the sequence selectivity of the protocol was confirmed. A fluorescence intensity profile plot across the microchannels is shown in Figure 3C. In this plot, the outer half of both channels shows significant fluorescence from the bulk F-SA solution. The inner half of both channels shows slight fluorescence, which is adopted as a background level.

Figure 4 shows the time course of signals generated by the LFDA with various concentrations of the target miRNA. The signals began to rise from the initial value (F-SA bulk fluorescence) at some point, depending on the target concentrations. Generally, a higher concentration caused an earlier rise. The blank sample showed the latest rise, typically after an elapsed time of 5 min, which was probably initiated by nonspecific adsorption of F-SA or B-anti-SA. Once the signals rose, they grew exponentially at similar rates. As a result, higher signals were obtained with higher target concentrations. One potential drawback of the power-free microchip is that its flow rate diminishes with time. We have previously investigated this effect, and found that the flow rate diminished by 35% in first 8 min [35]. Nevertheless, in general, the flow rate has only little effect on the solute adsorption onto the surface. Specifically, the adsorption rate is affected by a factor of cubic root of the flow rate at most [41]. Therefore, the decrease in flow rate of the power-free microchip is unlikely to cause a serious problem in this application.

A parameter for the calibration curve was defined as the ratio of signal over blank reference at the elapsed time when the blank reference in the right channel started to rise by nonspecific binding (Figure 5). Three data values were basically used to plot each average and standard deviation. (Five data were taken only for zero concentration.) The data points of the calibration curve were fitted with a four-parameter logistic function [42]: *y*  =  *d* + (*a* – *d*)/[1+ (*x/c*)*^b^*], where *a*, *b*, *c* and *d* are fitting parameters. These parameters were optimized by nonlinear least-square regression weighted by the reciprocal of the square of standard deviation of each datum point. The LOD was evaluated by the 3σ criterion. Namely, the LOD was calculated as the concentration (*x*) which gives *y* higher than the *y*-intercept of the fitting function (*a*) by three times the standard deviation of the zero-concentration data. This *y* value is indicated in Figure 5 as “3σ line.” As a result, we obtained an LOD of 0.5 pM, which corresponds to 0.25 amol, because we consumed a sample volume of 0.5 µL. Recently, Zhou et al. compared LOD of some existing miRNA analysis methods in terms of number of molecules (Table 1 in [43]), ranging from 0.2 zmol of qPCR to 1 fmol of blotting. Among them, the LOD obtained in this study can be evaluated as a moderate value. However, most of the existing methods take more than a few hours. This study is the first achievement of this level of LOD for miRNA in 20 min. Therefore, we have proven that the combination of a power-free microfluidic device and LFDA is effective for the simple, rapid, and sensitive detection of miRNA.

### Conclusions

This paper reports a new method for easy, rapid and highly sensitive miRNA detection with an LOD at the sub-attomole level by adopting LFDA using the power-free PDMS microfluidic device. The target miRNA was efficiently detected by sandwich hybridization followed by LFDA. Furthermore, unlabeled miRNA was successfully detected in 20 min without a PCR process or enzymatic amplification. The LOD was 0.5 pM from a 0.5 µL sample solution, which corresponds to 0.25 amol. Thus, the LOD has been improved by 3 orders of magnitude compared to that of our previous study [30] without spoiling the merits of rapidity and small sample volume.

In our previous study, miRNA detection by sandwich hybridization with the same construct was proven to be capable of multiple sequence detection from the same sample solution [30]. This capability strongly supports the fact that miRNA detection by this device is a promising technology for cancer diagnosis because each cancer is considered to have only a few number of corresponding marker miRNAs, which may exclude the need for screening hundreds of sequences by a conventional array chip. For practical application, the optical and electrical equipment for signal output should be miniaturized or combined with existing electronic devices [44]. These challenges might effectively demonstrate the potential of this method for commercial production. Rapidness, simple operation, small required sample volume, and portability of the device are ideal advantages for point-of-care cancer diagnosis. Therefore, further study might contribute to improvement of the healthcare environment even in resource-poor environments, such as found in developing countries, and may have both an industrial and societal impact on global health.

## Materials and Methods

### PDMS Fabrication

First, the PDMS part with the recessed patterns of the microchannels was fabricated as described elsewhere [33]. A negative master for the molding of PDMS was fabricated on a silicon wafer with an ultrathick photoresist (SU-8 25, MicroChem, Newton, MA). The surface of the master was passivated by exposure to CHF_3_ plasma in a plasma etcher (RIE-10NR, Samco International, Kyoto, Japan). A prepolymer of PDMS (Sylgard 184, Dow Corning, Midland, MI) was cast onto the master with a frame for holding the prepolymer and degassed in a vacuum chamber for more than 20 min. After incubation for 1 h at 65°C, the cured PDMS part was peeled off from the master, and through-holes for the waste reservoir and inlet holes were punched using a metal pipe. Two types of PDMS microchannels were used in this study: the first was a single channel for the selective patterning of the aminated probe DNA and the second was a pair of Y-shaped channels for miRNA detection. Both of the PDMS microchannels were fabricated and mounted onto the glass plate to form microfluidic channels 100 µm in width and 25 µm in height.

### Probe DNA Patterning on Glass Plate

Patterning of the aminated probe DNA on a glass surface was carried out as follows. First, glutaraldehyde (Wako) was incubated on an aminated glass plate (SD00011, 25.4 × 76.2 mm, Matsunami Glass Ind., LTD) at 37°C for 2 h. Next, the single channel PDMS described above was mounted onto the glass plate and degassed in a vacuum chamber at 10 kPa for 40 min. Amino-labeled DNA (Oligonucleotide, Europhins operon) was injected into the channel by power-free pumping and incubated at 37°C for 30 min. Finally, the PDMS microchannel was detached from the glass plate in a stopping buffer (25 mM Tris, 0.05% Tween20). The glass plate was washed by ultrasonication, using both the stopping buffer and deionized water to remove remaining DNA on the surface.

### MicroRNA Detection

The power-free microfluidic device was prepared by attaching the Y-shaped PDMS channels onto the probe DNA-patterned glass plate. The device was degassed in a vacuum chamber at 10 kPa for 40 min beforehand. A blocking buffer was prepared by dissolving a commercial product (Blocking solution #1585762, Roche Diagnostics, Basel, Switzerland) at a concentration of 1% (w/v) with 0.02% (w/v) sodium dodecyl sulfate in 5× saline-sodium citrate buffer containing 0.05% Tween 20. The protocol for target miRNA detection is summarized in Figure 2, and detailed in the figure legend.

### Data Acquisition and Analysis

During the LFDA, the intersections of immobilized probe DNA and microchannels were observed by an inverted fluorescence microscope (TE2000-U, Nikon, Tokyo, Japan) equipped with a 100 W mercury lamp, a dichroic mirror block (excitation: 465–495 nm; emission: 515–555 nm), a 10× objective lens, and a cooled charge-coupled device (CCD) camera (CoolSNAP HQ2, Photometrics, Tucson, AZ). Images were captured by the CCD camera every minute and fluorescence intensities were analyzed by image analysis software (Image J 1.38×, National Institutes of Health, USA).
